# Molecular Detection of *Plasmodium malariae/Plasmodium brasilianum* in Non-Human Primates in Captivity in Costa Rica

**DOI:** 10.1371/journal.pone.0170704

**Published:** 2017-01-26

**Authors:** Alicia Fuentes-Ramírez, Mauricio Jiménez-Soto, Ruth Castro, Juan José Romero-Zuñiga, Gaby Dolz

**Affiliations:** 1 Maestría en Enfermedades Tropicales, Posgrado Regional en Ciencias Veterinarias Tropicales (PCVET), Universidad Nacional (UNA), Campus Benjamín Nuñez, Barreal de Heredia, Costa Rica; 2 Escuela de Medicina Veterinaria, UNA, Campus Benjamín Núñez, Barreal de Heredia, Costa Rica; Institute of Tropical Medicine, JAPAN

## Abstract

One hundred and fifty-two blood samples of non-human primates of thirteen rescue centers in Costa Rica were analyzed to determine the presence of species of *Plasmodium* using thick blood smears, semi-nested multiplex polymerase chain reaction (SnM-PCR) for species differentiation, cloning and sequencing for confirmation. Using thick blood smears, two samples were determined to contain the *Plasmodium malariae* parasite, with SnM-PCR, a total of five (3.3%) samples were positive to *P*. *malariae*, cloning and sequencing confirmed both smear samples as *P*. *malariae*. One sample amplified a larger and conserved region of 18S rDNA for the genus *Plasmodium* and sequencing confirmed the results obtained microscopically and through SnM-PCR tests. Sequencing and construction of a phylogenetic tree of this sample revealed that the *P*. *malariae/P*. *brasilianum* parasite (GenBank KU999995) found in a howler monkey (*Alouatta palliata*) is identical to that recently reported in humans in Costa Rica. The SnM-PCR detected *P*. *malariae/P*. *brasilianum* parasite in different non-human primate species in captivity and in various regions of the southern Atlantic and Pacific coast of Costa Rica. The similarity of the sequences of parasites found in humans and a monkey suggests that monkeys may be acting as reservoirs of *P*.*malariae/P*. *brasilianum*, for which reason it is important, to include them in control and eradication programs.

## Introduction

Malaria is a vector-borne disease caused by protozoa of the *Plasmodium* genus, which are transmitted to human beings and to non-human primates through the bite of female blood-sucking mosquitoes of the genus *Anopheles* [1, 2]. Malaria parasites tend to be specific to each species, however, there are species of the parasite which infect both persons and monkeys, and cannot be differentiated morphologically, genetically or immunologically [3].

The presence of approximately 33 species of this parasite [4, 5] has been reported in non-human primates. In monkeys of the New World, the main species reported are *Plasmodium brasilianum* and *Plasmodium simium* [6], which cannot be differentiated from *Plasmodium malariae* and *Plasmodium vivax*, respectively [7–9]. The presence of *P*. *simium* has been reported only in three monkey species in Brazil [8, 10], while *P*. *brasilianum* has been described in approximately 35 monkey species, mostly among members of the *Cebidae* and *Atelidae* families in Central and South America [4, 7, 8]. It is still unknown if under natural conditions non-human primates carrying *Plasmodium* could develop malaria, or if they only act as reservoirs of the parasite [10]. However, in those animals that have been subjected to experimental splenectomy, it has been observed that infection by *Plasmodium* spp. may cause weakness and malaria, with fever, anemia, jaundice and kidney failure [1, 6, 11].

The species of *Plasmodium* that cause infection in non-human primates are of great interest, as they may be transmitted to human beings naturally or by accident [7]. Human invasion of the natural habitats of non-human primates, and their contact with these animals in captivity, gives rise to the possibility of acquiring natural infections, although the vectors are different [3, 12]. In Asia, it has been shown that some species of mosquitoes of the genus *Anopheles* (*Anopheles latens* and *Anopheles cracens*) are highly zoophilic, feeding on monkeys in tree canopies as well as on humans at ground level, enabling human-to-human, monkey-to-monkey, human-to-monkey and monkey-to-human transmission of *Plasmodium knowlesi* [3]. In contrast, vectors of natural malaria in humans and non-human primates in South America seem to be the same, as is the case for *Anopheles Kerteskia cruzii*. It is almost the only species found to naturally carry and transmit *P*. *vivax*/*P*. *simium* and *P*. *malariae/P*. *brasilianum* to man and monkeys in Brazil [13, 14].

In Costa Rica, the *P*. *brasilianum* species was reported in 2006 in six free-living howler monkeys, using extended smears stained with Giemsa [15]. The first four cases since 1959 of *P*. *malariae* were recently diagnosed in persons through molecular analysis (real-time PCR); sequencing confirmed the results, and identified them as *P*. *malariae/P*. *brasilianum* [16]. It remains to be determined whether *P*. *malariae* is a re-emerging species in the country, if it has been present during all of this time and had not been microscopically detected, or if it had been confused with *P*. *vivax*, given the morphological similarity of early stages of the parasites of these two species [16]. The objective of the present study was to determine and characterize, using molecular techniques, the *Plasmodium* species present in blood samples of non-human primates living in captivity, to determine its similarity to species present in cases of humans in Costa Rica.

## Materials and Methods

### Ethics statement

The procedures applied in this specific study to the non-human primates were approved by an ethics committee (FCSA-EMV-CBAB-005-2014, Animal Welfare and Bioethics Board, School of Veterinary Medicine, Universidad Nacional, Heredia); adhered to the legal requirements of Costa Rica and to the American Society of Primatologists (ASP) Principles for the Ethical Treatment of Non-Human Primates. All centers that participated were under the supervision of the Ministry of Environment and Energy from Costa Rica, and had a biologist regent and a veterinary medical adviser. The centers conditioned their participation to that the names of their establishments were not mentioned in the investigation. The non-human primates were living in wire mesh cages following the recommendations of the Code of Practice for the Welfare of Privately Kept Non-Human Primates (https://www.gov.uk/government/uploads/system/uploads/attachment_data/file/218679/primate-cop.pdf).

### Type of study and sample size

A cross-sectional descriptive study was carried out to detect *Plasmodium* species in non-human primates in captivity in wildlife Rescue Centers and zoos. All the Centers that were willing to participate were included in the study. A simple random probabilistic sample was carried out in which all the monkey species present in these facilities were included. Primates were immobilized using sedatives. The anesthesia protocol consisted of Ketamine (5–20 mg/kg), combined with Diazepam (0.5–2 mg/kg), administered intramuscularly, monitoring vital parameters.

Information about sex and age was collected for these animals. Each animal underwent an objective clinical examination in which the following parameters were evaluated to determine the overall health status of the animals: body condition, coat condition, color of mucous membranes, status of lymph nodes, and hydration status. Animals were classified as being in normal clinical condition when all parameters were normal, in fair condition when one to three parameters presented any abnormality, and in bad condition when four or five parameters were abnormal. Finally, blood was extracted from the basilic, cephalic or femoral vein (approximately 1–2 ml). Samples were collected in EDTA tubes and transported to the laboratory at 4°C, where they were stored at 20°C until analyzed (S1 Table).

### Microscopic analysis

A thick blood smear and a thin extended smear fixated with methanol on a glass slide were made for each sample; each slide was stained with Giemsa dye for 15 minutes, and was microscopically examined using a 100x immersion objective lens to identify hemoparasites. A sample was classified as positive by microscopy whether *Plasmodium* spp. was observed either in the thick blood smears or thin extended smears in 500 leukocytes. Positive slides were sent to the National Reference Laboratory of Parasitology, of the Costa Rican Institute for Research and Education in Nutrition and Health (whose initials in Spanish are CNRP-INCIENSA) in Cartago, Costa Rica, to be confirmed.

### Molecular analysis

The DNA was extracted from total EDTA blood, using the commercial QIAGEN DNeasy Blood & Tissue kit, following the protocol established by the manufacturer. Detection and identification of the *Plasmodium* species were carried out using semi-nested multiplex polymerase chain reaction (SnM-PCR) which amplifies a region of the 18S ribosomal DNA, previously described by Rubio et al. [17], with some modifications in timing and temperature. The mix for the first reaction consisted of 14 μl of Dream Taq^TM^ PCR Master Mix (Fermentas®), 1 μl (20 pmol/μl) of PLF primer, 1 μl (20 pmol/μl) of UNR primer, 1 μl (20 pmol/μl) of HUF primer (Table 1), 5 μl of DNA, and reaction was completed with nuclease-free H_2_0 (Fermentas®) until a final volume of 25 μl was reached. The amplification protocol was the following: 5 minutes of initial denaturing at 94°C, 40 repeated denaturing cycles (94°C for 1 minute), alignment (62°C for 1 minute), extension (72°C for 90 seconds), and a final extension temperature of 72°C for 10 minutes. A second reaction was carried out with specific primers to detect species within this genus, using: 5 μl of the product amplified in the previous PCR, 13 μl of Dream Taq^TM^ PCR Master Mix (Fermentas®), 1 μl (20 pmol/μl) of PLF primer, 1 μl (20 pmol/μl) of MAR primer, 1 μl (20 pmol/μl) of FAR primer, 1 μl (20 pmol/μl) of VIR primer, to amplify *P*. *malariae*, *P*. *falciparum and P*. *vivax*, respectively [17] (Table 1). Each of these reactions was completed with nuclease-free H_2_O (Fermentas®) until a final volume of 25 μl was reached. The amplification conditions were the following: 5 minutes of initial denaturing at 94°C, 35 repeated cycles of denaturing (94°C for 30 seconds), alignment (62° for 30 seconds), extension (72°C for 1 minute), and a final extension temperature of 72°C for 10 minutes.

**Table 1:** Sequences of primers used in the SnM-PCR and in the 18S rDNA PCR for genera *Plasmodium*.

PCR	Primer	Sequence	Specificity	Product Size	Reference
**SnM-PCR First round**	PLF	5’-AGTGTGTATCCAATCGAGTTTC-3’	*Plasmodium*	783-821bp*	[17]
	HUF	5’-GAGCCGCCTGGATACCGC-3’	Mammals	231 bp^++^	
	UNR	5’-GACGGTATCTGATCGTCTTC-3’	Universal		
**SnM-PCR Second round**	PLF	5’-AGTGTGTATCCAATCGAGTTTC-3’	*Plasmodium*		
	MAR	5’-GCCCTCCAATTGCCTTCTG-3’	*P*. *malariae*	269 bp	
	FAR	5’-AGTTCCCCTAGAATAGTTACA-3’	*P*. *falciparum*	369 bp	
	VIR	5’-AGGACTTCCAAGCCGAAGC-3’	*P*. *vivax*	499 bp	
**18S rRNA PCR**	rPLU5	5’-CCTGTTTGTTGCCTTAAACTTC-3’	*Plasmodium*	1050 bp	[18]
	rPLU6	5’-TTAAAATTGTTGCAGTTAAAACG-3’	*Plasmodium*		

*Size depending on the species, *P*. *malariae* (821 bp), *P*. *falciparum* (787 bp), *P*. *vivax* (783 bp).

^++^ In the first reaction, the UNR-HUF primers form a product of 231 bp which is used as an individual positive control, and must be present in all the samples except the negative control.

The expected size of the amplification fragments of SnM-PCR was 269 bp for *P*. *malariae*, 395 bp for *P*. *falciparum*, 499 bp for *P*. *vivax*. Furthermore, positive samples detected in the SnM-PCR were cloned in the pCR2.1 vector using a TOPO TA cloning kit, according to the protocol recommended by the manufacturer (Invitrogen, Carlsbad, CA).

Finally, SnM-PCR positive samples were subjected to a second PCR, that amplified a larger (~1050 pb) and conserved region of 18S rDNA to detect parasites at the level of the *Plasmodium* genus. This amplification was performed as described by Leclerc et al. [18], with modifications in timing and temperature as follows: 14 μl of Dream Taq^TM^ PCR Master Mix (Fermentas®), 1 μl (20 pmol/μl) of rPLU5 primer, 1 μl (20 pmol/μl) of rPLU6 primer (Table 1), 5 μl of DNA, completed with nuclease-free H_2_0 (Fermentas®) until a final volume of 25 μl was reached. The amplification was carried out as follows: 5 minutes of initial denaturing at 95°C, 35 repeated cycles of denaturing (95°C for 1 minute), alignment (50°C for 1:30 minutes), extension (72°C for 1 minute), and a final extension temperature of 72°C for 5 minutes. In the two PCRs, nuclease-free H_2_O (Fermentas®) was used as a negative control, and *P*. *malariae*, *P*. *vivax and P*. *falciparum* DNA provided by CNRP-INCIENSA were used as positive controls.

The PCR products and clones containing target inserts were observed through electrophoresis in agarose gel at 1.5%, in TBE 1X (Tris base, boric acid, EDTA, pH8, 0.5M) and stained with GelRed^TM^ Nucleic Acid Gel Strain dye. GenRuler 100 bp DNA Ladder Plus (Fermentas®) was used as a molecular weight marker. Additionally, plasmid DNAs were purified with a QIAprep Spin Miniprep Kit (Qiagen, Valencia, CA).

### Sequencing and phylogenetic tree

The positive products of both PCRs and plasmid DNA templates were sent to Macrogen (Seoul, Korea) to be sequenced to confirm the results. Plasmid DNA from two clones per sample were selected for sequencing with the universal M13 primers. Sequences were manually edited with the BioEdit Sequence Aligment Editor® program [19], and compared against the National Center for Biotechnology Information (NCBI) database using BLAST. Sequences were aligned in MUSCLE [20] with pre-defined parameters. Phylogenetic analyses were carried out using the Molecular Evolutionary Genetics Analysis (MEGA) program, version 6.0, and the phylogenetic tree was constructed using the Maximum Likelihood method of a region of 860 pb of 18S rDNA. The Tamura-Nei model was used to measure evolutionary distances and the relationship of the clades was determined through a bootstrap value of 1,000 replications. Other sequences in the same region of *Plasmodium* spp. available in the GenBank data base with access numbers HQ283216 (*P*. *falciparum*), AY579418 (*P*. *vivax*), AY579417 (*P*. *knowlesi*), AY579415 (*P*. *simium*), AF130735, KT266778, KC906723 (*P*. *brasilianum*), AF145336 and KJ934251 (*P*. *malariae*, the last reported in Costa Rica) were used. *Theileria* spp. (AF162432) was used as an external group.

## Results

### Population analyzed and sample collection

**Fig 1:**
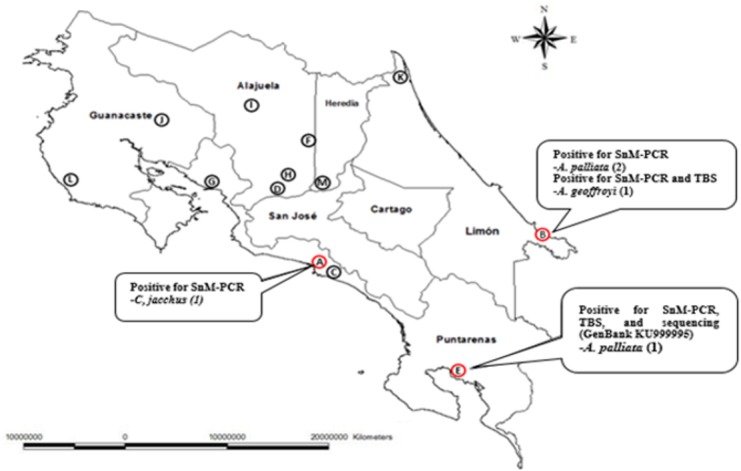
Geographic locations of rescue centers and positive cases of *P*. *malariae* detected through TBS, PCR and sequencing.

Thirteen animal rescue centers were visited between April and November, 2015, in which a total of 152 monkey blood samples were collected (Fig 1). The study included four native species of monkeys of Costa Rica: *Saimiri oerstedii* (16), *Alouatta palliata* (32), *Ateles geoffroyi* (48), *Cebus imitator* (39), and 17 monkeys of the non-native species *Callithrix jacchus* (marmosets). The number of non-human primates analyzed by species and by rescue center is presented in Table 2. The description of the population of monkeys from which the samples were taken in the rescue centers, based on the parameters evaluated in the clinical evaluation, is detailed in Table 3. The species of primates more frequently found in captivity were *A*. *geoffroyi* (31.6%), and *C*. *imitator* (25.6%). The population of female monkeys in captivity was larger (55.9%). Most of the monkeys in captivity were adult (70.4%) and showed normal physical condition (82.2%).

**Table 2:** Number of non-human primates analyzed by species and by rescue center.

Species	Rescue Center													Total
	A	B	C	D	E	F	G	H	I	J	K	L	M	
***S*. *oerstedii***	7/7	-	-	-	3/3	-	-	6/6	-	-	-	-	-	16/16
***A*. *palliata***	4/4	9/11	-	5/6	3/4	-	-	5/6	-	4/6	-	1/6	1/1	32/44
***A*. *geoffroyi***	-	5/6	5/6	4/5	3/5	5/5	8/9	6/6	7/9	4/5	1/1	-	-	48/57
***C*. *imitator***	3/3	3/4	2/2	3/4	1/1	6/7	-	6/7	11/12	2/2	2/2	-	-	39/44
***C*. *jacchus***	17/18	-	-	-	-	-	-	-	-	-	-	-	-	17/18
**Total**	31/32	17/21	7/8	12/15	10/13	11/12	8/9	23/25	18/21	10/13	3/3	1/6	1/1	**152/179**
**(%)**	(96.8)	(80.9)	(87.5)	(80.0)	(76.9)	(91.6)	(88.8)	(92.0)	(85.7)	(76.9)	(100.0)	(16.6)	(100.0)	**(84.9)**

**Table 3:** Description of the monkey population analyzed in 13 rescue centers in Costa Rica, based on clinical evaluation.

Characteristics	Stratum	Monkeys	(%)
		n=152	
**Sex**	Female	85	55.9
	Male	67	44.1
**Age**	Adult	107	70.4
	Young	45	29.6
**Body condition**	Obese	5	3.3
	Normal	125	82.2
	Thin	20	13.2
	Cachectic	2	1.3
**Fur condition**	Good	147	96.7
	Bad	5	3.3
**Alopecia**	Yes	12	7.9
	No	140	92.1
**Ectoparasites**	Yes	0	0
	No	152	100
**Mucous membranes**	Normal	148	97.4
	Congestive	1	0.6
	Jaundiced	0	0
	Pale	3	2.0
	Cyanotic	0	0
**Hydration**	Normal	146	96.1
	Slight dehydration	5	3.3
	Moderate dehydration	1	0.6
	Severe dehydration	0	0
**Lymphonodes**	Normal	149	98.0
	Enlarged	3	2.0
**Previous diseases**	Yes	23	15.1
	No	122	80.3
**Treatments applied**	Unknown	7	4.6
	Yes	35	23.0
	No	112	73.7
	Unknown	5	3.3
**Veterinary evaluation in the rescue center**	Yes	149	98.0
	No	3	2.0
**Deworming treatments**	Yes	140	92.1
	No	3	2.0
	Not yet (because of age)	9	5.9

### Thick blood smear

Of the 152 blood samples analyzed with thick blood smears and thin extended smears, two were found to be positive, in which the parasite’s trophozoites and schizonts were observed and identified as *Plasmodium malariae/Plasmodium brasilianum*. Of these two samples, only the E1 sample was confirmed by the CNRP-INCIENSA (Table 4, Fig 1).

**Table 4:** Descriptive analysis of animals that were positive in thick blood smear and SnM-PCR for *P*. *malariae*.

Rescue Center	Sample	Species	Sex	Age	BC	FC	Muc	LN	PD	TBS	SnM-PCR
**A**	A1	*C*. *jacchus*	M	Y	N	G	N	N	No	NPO	*P*. *malariae*
**B**	B1	*A*. *palliata*	F	Y	N	G	N	N	No	NPO	*P*. *malariae*
**B**	B2	*A*. *palliata*	M	Y	N	G	N	N	No	NPO	*P*. *malariae*
**B**	B3	*A*.*geoffroyi*	F	Y	N	G	N	N	Lost an eye	*P*. *malariae*^TR^	*P*. *malariae*
**E**	E1	*A*. *palliata*	F	Y	T	G	N	N	Lost an eye	*P*. *malariae*^TR, SC^	*P*. *malariae*

M: male, F: female, Y: young, BC: body condition, N: normal, T: thin, FC: fur condition, G: good, Muc: mucous membranes, LN: lymphnodes, PD: previous diseases, NPO: no parasites observed, TR: trophozoites, SC: schizonts.

### SnM-PCR, cloning of SnM-PCR amplicons, and PCR results for genera

**Fig 2:**
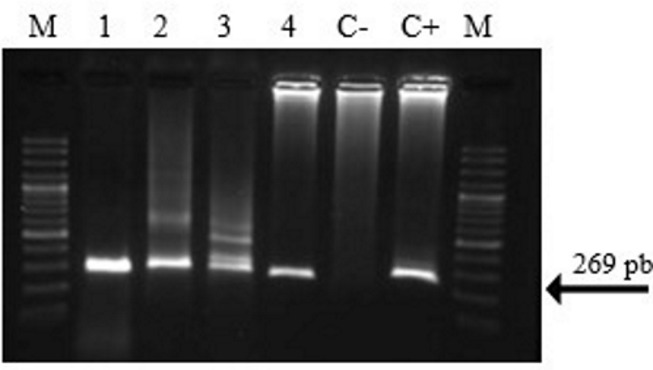
Electrophoresis in agarose gel of the products of the SnM-PCR for *Plasmodium* species. (M) molecular weight marker, (1) sample A1, (2) sample B1, (3) sample B2, (4) sample E1, (C -) negative control, (C+) positive control of *P*. *malariae*. Sample B3 is not shown.

Of the 152 animals studied, a total of five samples were detected by SnM-PCR to be positive for *P*. *malariae/P*. *brasilianum* (Fig 2). Two of these animals had been previously detected as positive in thick blood smears analyses. The descriptive analysis of the five positive samples is presented in Table 4, while the geographic location of the rescue centers with the positive cases are presented in Fig 1. A total of two amplicons of the SnM-PCR were successfully cloned (samples B3 and E1). The five samples were analyzed with the second PCR, only one sample (E1) was amplified (Fig 1, Table 4).

### Results of sequencing and phylogenetic tree

**Fig 3:**
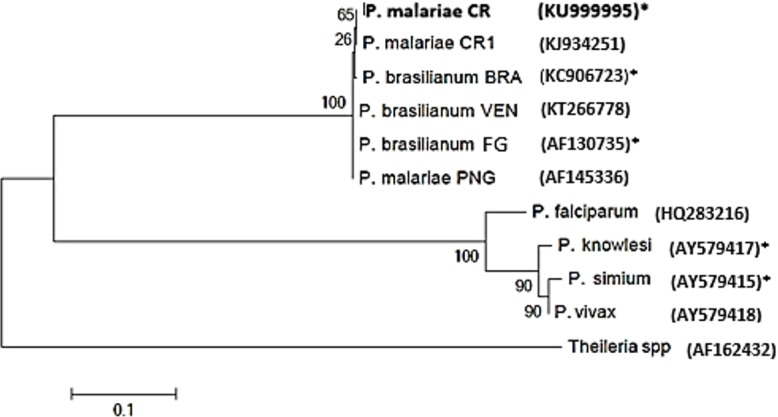
Phylogenetic tree constructed based on rDNA 18 S sequences of *P*. *malarie/P*. *brasilianum*. Maximum Likelihood and Tamura-Nei were used as evolutionary and genetic distance models, respectively. The re-sampling (bootstrap) analysis (with a percentage of 1,000 replications) is represented by numbers in each of the nodes. Countries are coded: (CR = Costa Rica, BRA = Brazil, VEN = Venezuela, FG = French Guiana, PNG = Papua New Guinea).*Sequences isolated from non-human primates. The remaining sequences were isolated from humans.

The two clones from samples B3 and E1 were sequenced (~250 bp) and deposited in the GenBank database with the accession numbers KY246307 and KY246308, respectively. The B3/E1 sequences were (99%/100%) identical to *Plasmodium malarie* isolate JGS993 from India (KU510228). The larger sequence obtained from the E1 sample (860 bp) was deposited in the GenBank (KU999995) and showed 100% nucleotide identity with *P*. *malariae/P*. *brasilianum* (GenBank KJ934251) reported in human cases that occurred in Costa Rica in 2012–2013 [16] (Fig 3).

## Discussion

This work is the first cross-sectional study which combines diagnostic thick blood smear and molecular biology techniques (PCR and sequencing), to detect parasites of the *Plasmodium* genus in non-human primates in captivity in Costa Rica. Given that 84.9% of the population of monkeys living in captivity in 13 rescue centers were analyzed, and these centers receive animals from different parts of the country, this work is based on a strong representative sample of the population studied, which provides internal validity [21]. In this study, the *P*. *malaria*e/*P*. *brasilianum* parasite was found in three non-human primate species (*A*. *palliata*, *A*. *geoffroyi and C*. *jacchus*), which is consistent with what was reported by other researchers, who also found *P*. *malariae/P*. *brasilianum* in free-living monkeys in Costa Rica [15].

The general condition of non-human primates in captivity was determined as good; fair condition of their fur was found in only 3.3% of the total number of animals examined. The howler monkey and spider monkey that tested positive for *P*. *malariae/P*. *brasilianum* in thick blood smears, SnM-PCR, cloning, and sequencing analyses were both young females that had lost an eye, presumably in fights with other monkeys. The howler monkey was found wounded in the forest, and taken to the rescue center for medical assistance; at the time when the sample was taken, it had been in the rescue center for a month. The spider monkey lived in a rescue center, where juveniles are rehabilitated by the crew taking and staying with them in the wild during the daytime. Once they become grown adults they are released. Positive cases were not found among any of the other animals analyzed in the same center (N = 10), located in the South Pacific, whereas two additional howler monkeys were determined positive to SnM-PCR in the rescue center located in the South Atlantic (N = 17), although sequencing of these samples was not achieved. It is possible that the howler monkey in the South Pacific and the spider monkey in the South Atlantic acquired the parasite when it was living freely or moving freely in the woods, respectively, and a wild cycle is probably taking place in those sites for the parasite, which would be important to confirm in further studies [15].

Our results are consistent with those reported by other authors about the low sensitivity of the thick blood smear compared to PCR [22–24]; a total of five (3.3%) of 152 samples tested positive using SnM-PCR, while only two (1.3%) tested positive using the thick blood smear. These data also reveal a low prevalence of parasites of the *Plasmodium* genus in monkeys analyzed in rescue centers in Costa Rica, which is consistent with investigations in other countries such as Brazil and French Guiana, where authors have reported prevalence’s below 6% in non-human primates in captivity [4, 25, 26].

Semi-nested multiplex polymerase chain reaction (SnM-PCR) testing yielded 5 positive results for the *P*. *malariae/P*. *brasilianum* species, which is consistent with what is described in the literature–that it is the most prevalent *Plasmodium* species in the New World monkeys [4, 10]. Three of these five parasite carriers were confined in a rescue center in the Caribbean zone, where the majority of malaria cases in humans in Costa Rica have occurred [27, 28]. Chinchilla et al. also reported the presence of the parasite in free-living howler monkeys in this region [15]. The other monkeys who were carriers of the parasite were living in rescue centers in the Central Pacific and South Pacific zones. The places in which the animals were living in these rescue centers had ideal environmental characteristics, such as altitude and temperature for the survival of the etiological agent and the transmitting vector [27, 28]. Sequencing of the SnM-PCR amplicons confirmed the presence of *P*. *malariae/P*. *brasilianum* in two species (*A*. *palliata* and *A*. *geoffroyi*) and in two different rescue centers located, one at the Pacific and the other at the Atlantic coast. Finally, this study reports a possible non-native monkey species *Callithrix jacchus* infected with *P*. *malariae/P*. *brasilianum*, but confirmation by sequencing is still needed. However, these findings are in accordance with Deane, who reported the simian malaria as widely spread in Brazil, detecting *P*. *brasilianum* in 28 species of Cebidae and in one species of Callitrichidae [13].

The sequences of the SnM amplicons were similar to parasites isolated from humans in India, whereas sequencing of the 18S rDNA region determined that the *P*. *malariae/P*. *brasilianum* parasite found in the monkey in the South Pacific area is identical to the parasites recently found in humans, which had not been detected in Costa Rica since 1959 [16]. Both PCRs (SnM and 18s rDNA) amplified different fragments of the 18S rDNA region.

Studies based on analyses of the sequence of the 18S rDNA gene of *P*. *brasilianum and P*. *malariae* show that the similarity between both species is greater than 99%, and that they are only different in a simple nucleotide polymorphism [29], supporting previous results [30]. In addition, analysis of sequences of *Csp* [30, 31] and *Msp-1* [31] protein encoding genes also corroborated that *P*. *brasilianum* and *P*. *malarie* are genetically indistinguishable.

Presently, the two parasites are perceived as variants of the same species, which have become increasingly specialized in different hosts. Therefore, when they are found in monkeys, they are called *P*. *brasilianum*, and when they are found in humans, they are called *P*. *malariae* [29]. However, comprehensive studies needed to be carried out, to finally and definitively establish whether both *P*. *malariae* and *P*. *brasilianum* are the same or distinct species [32].

In optimal conditions, where close contact exists between monkeys and humans, and a competent mosquito vector is present, the parasites of quartan malaria are able to cross borders between hosts, producing an anthropozoonosis [13, 26, 29, 33]. The present results provide evidence that non-human primates may be acting as reservoirs for parasites of the genus *Plasmodium*, making it difficult to eradicate malaria in the country.

The consequences of this situation are important, and the emergence of an alternative host of the parasite requires special attention by health authorities [26, 34]. Studies should be carried out to determine the species of vectors involved in the transmission of *P*. *malariae/P*. *brasilianum* in monkeys and humans in the places where parasite-carrying monkeys have been found. Costa Rica is currently in the phase of pre-elimination of the disease [28], and it is therefore critical to include non-human primates in the disease’s control and eradication programs. The results of three samples were not confirmed by sequencing, this could be either that amplicons were denatured or that they represent *P*. *malariae/P*. *brasilianum* variants, which should be investigated in future studies [14].

## Conclusions

The *P*. *malariae/P*. *brasilianum* parasite was found in different species of non-human primates in captivity, and in different regions of the Southern part of Costa Rica, on both the Pacific and Atlantic coasts. The similarity of the sequences of the parasites found in humans and one monkey suggests that monkeys may be acting as reservoirs of *P*.*malariae/P*. *brasilianum*, for which reason it is important to include them in the control and eradication programs.

## Supporting Information

S1 Table Complete database of 152 primates non-humans analyzed in 13 rescue centers in Costa Rica. (XLS)
